# Prevalence and Specificity of Red Blood Cell Alloantibodies in Patients in a Tertiary Care Center in Meghalaya, India

**DOI:** 10.7759/cureus.86347

**Published:** 2025-06-19

**Authors:** Khumanthem M Devi, Lutika Nepram, Ranendra Hajong, Debdutta Bhattacharyya

**Affiliations:** 1 Transfusion Medicine and Blood Centre, North Eastern Indira Gandhi Regional Institute of Health and Medical Sciences (NEIGRIHMS), Shillong, IND; 2 Surgery, North Eastern Indira Gandhi Regional Institute of Health and Medical Sciences (NEIGRIHMS), Shillong, IND

**Keywords:** alloantibody, alloimmunization, autoantibody, pre-transfusion testing, unexpected antibody

## Abstract

Introduction

Red blood cell (RBC) alloimmunization complicates transfusion therapy by making compatible units scarce and increasing the likelihood of hemolytic transfusion reactions, particularly in multi-transfused or sensitized patients. Data from northeastern India remain scarce. This study aims to evaluate the prevalence and specificity of RBC alloantibodies and their clinical associations at a tertiary care center in Meghalaya.

Methods

We conducted a retrospective, cross-sectional review in the Department of Transfusion Medicine and Blood Centre, North Eastern Indira Gandhi Regional Institute of Health and Medical Sciences, Shillong. From March 2021 to February 2024, we screened every patient with an RBC transfusion request using an antibody screening cell panel and, when necessary, performed antibody identification. We retrieved clinical and transfusion histories from laboratory records and used chi-square tests to examine associations with alloantibody formation.

Results

Although the overall frequency of alloantibody development was low, certain patterns emerged, most notably higher prevalence in women and transfusion-dependent patients. Women and patients with prior transfusions developed alloantibodies more often than their counterparts. Anti-E, the most common antibody, dominated the Rhesus system findings, and antibodies in the Kidd system were also frequent. Antenatal patients formed the largest subgroup with alloantibodies.

Conclusions

Routine antibody screening and precise identification should remain central to pre-transfusion testing, particularly for transfusion-dependent individuals and pregnant patients. Our region-specific data support targeted antigen-matching strategies and reinforce the need for robust transfusion policies to enhance patient safety and optimize blood-product use.

## Introduction

Blood transfusion services are an essential component of modern health care. Red blood cell (RBC) transfusion offers significant benefits in various clinical settings, including anemia due to pregnancy, malignancy, and trauma, by maintaining oxygen delivery and hemodynamic stability [1]. The International Society of Blood Transfusion (ISBT) recognized 378 antigens, of which 345 are clustered within 43 blood group systems, and a few more were added later. The ABO blood group system includes antigens A, B, AB, and A1, as well as naturally occurring antibodies anti-A and anti-B which are present in human serum or plasma [2,3].

Minor blood group systems include, but are not limited to, Rhesus, MNS, P1PK, Lutheran, Kell, Lewis, Duffy, and Diego. Antibodies associated with these systems are called unexpected antibodies because they typically require immune exposure, such as pregnancy, transplantation, or prior blood transfusion, for development. These antibodies may be alloantibodies (i.e., when a person produces an antibody against an antigen they lack) or autoantibodies (i.e., when a person produces an antibody against an antigen they possess) [2-4].

The risk of RBC alloimmunization is a major concern in transfusion recipients. Given the genetic diversity of the Indian population, a higher prevalence of alloimmunization can be expected among transfusion recipients. Alloantibodies can lead to adverse transfusion reactions, including acute or delayed hemolytic transfusion reactions (HTRs), and may reduce the survival of transfused RBCs. Their presence complicates the identification of compatible blood units and can delay transfusion [3,5].

ISBT has identified nine blood group systems as clinically significant due to their association with HTRs and hemolytic disease of the fetus and newborn (HDFN). These include ABO, Rhesus, Kell, Duffy, Kidd, MNS, P, Lewis, and Lutheran systems. After ABO and Rhesus, the Kell, Kidd, Duffy, and MNS systems are particularly important, as exposure to these antigens may lead to the rapid destruction of transfused erythrocytes through immune mechanisms [6]. The prevalence of alloantibodies in different populations can predict the likelihood of finding compatible blood for a patient who has antibodies to RBCs and can be useful in preparing the most suitable negative blood group antigens that would be selected from the inventory [7-9].

While several studies have evaluated the prevalence of RBC alloantibodies across various regions of India, limited data are available for the state of Meghalaya. Therefore, this study aims to determine the prevalence and specificity of RBC alloantibodies among patients at the North Eastern Indira Gandhi Regional Institute of Health and Medical Sciences (NEIGRIHMS), Meghalaya, India.

## Materials and methods

Study design

This retrospective cross-sectional study was conducted in the Department of Transfusion Medicine and Blood Centre at NEIGRIHMS, Meghalaya, India, from March 2021 to February 2024. The NEIGRIHMS Scientific Advisory Committee approved the study, and ethical clearance was obtained from the Institute Ethical Committee, NEIGRIHMS (Project Number: NEIGR/IEC/M5/F9/2024).

Inclusion and exclusion criteria

Patients with RBC blood requisitions from various clinical departments had undergone antibody screening and were included in the study. Patient demographic data, blood group results, and antibody screening and identification records were obtained from the antibody screening register.

Patients receiving only platelet concentrate or fresh frozen plasma were excluded. Platelet concentrate and fresh frozen plasma were matched based on ABO and Rhesus blood groups only and antibody screening was not done routinely. Samples with positive autocontrols, indicative of autoantibodies, were excluded to focus exclusively on alloimmunization.

Crossmatching was performed for patients who tested negative for unexpected RBC antibodies, and compatible blood units were issued. Antibody identification was performed for patients who tested positive for unexpected alloantibodies, and corresponding antigen-negative blood units were selected. Crossmatching was then conducted using the antigen-negative units, and compatible units were issued accordingly.

Laboratory methods

Blood grouping, antibody screening, and antibody identification were performed using the Neo Iris fully automated immunohematology analyzer (Immunocor India Pvt. Ltd., New Delhi, India). ABO grouping was conducted using Immunocor reagents. Antibody screening was carried out using the Capture-R Ready-Screen three-cell panel (Immunocor), which includes R1wR1, R2R2, and rr cell types. These panels test for the following antigens: D, C, E, e, C^w^, K, k, Kp^a^, Kp^b^, Js^b^, Fy^a^, Fy^b^, Jk^a^, Jk^b^, Le^a^, Le^b^, P1, M, N, S, s, Lu^b,^ and Xg^a^. Antibody identification was performed using the Capture-R Ready-ID 14-cell panel (Immunocor), which includes D, C, c, E, e, C^w^, K, k, Kp^a^, Kp^b^, Js^b^, Fy^a^, Fy^b^, Jk^a^, Jk^b^, Le^a^, Le^b^, P1, M, N, S, s, Lu^a^, Lu^b,^ and Xg^a^. Autocontrol was performed by reacting the patient’s own RBC with their serum to detect autoantibodies. The antibody screening methods and identification were low ionic strength saline solution (LISS)-antihuman globulin (AHG)-based.

In complex cases, manual blood grouping was performed using antisera from Tulip Diagnostics Pvt. Ltd. (Goa, India). For patients with both autoantibodies and alloantibodies, glycine-hydrochloric acid (HCl)/ethylenediaminetetraacetic acid (EDTA) elution in conjunction with the adsorption technique was done to separate alloantibodies. Heat elution was done for ABO-related HDFN and for elution of IgM antibodies from red cells [10].

For patients with RBC autoantibodies, compatible units were provided if available. When no compatible unit could be located, the least incompatible units (i.e., those showing weaker reactions than the autocontrol) were selected and issued. In life-threatening emergency transfusion cases, we released the initial packed red blood cell (PRBC) units on the basis of ABO and Rh (D) grouping alone. We then performed antibody screening and full crossmatching using tubing segments from the issued units and from any additional PRBC units required for ongoing transfusion. If we detected incompatibility, we notified the treating clinician immediately and initiated appropriate management.

Statistical analysis

Frequencies of alloantibodies were calculated and expressed as absolute numbers and percentages. Data were analyzed using SPSS for Windows, Version 16.0. (SPSS Inc., Chicago, IL, USA), and graphical representations were created using Microsoft Excel 2007 (Microsoft Corporation, Redmond, WA, USA). The chi-square test (χ²) was used to assess the association between alloantibody formation and variables such as gender and history of previous transfusion. A p-value of < 0.05 was considered statistically significant.

## Results

Eight thousand six hundred fifty-seven patients were screened for red cell-directed antibodies during the study period. Unexpected or irregular RBC antibodies were detected in 74 patients (0.85%). Among these, 65 patients had alloantibodies, yielding an alloimmunization rate of 65/8,657 (0.75%), while autoantibodies were detected in 9/8,657 (0.10%) of patients. The mean age of patients with alloantibodies was 37.4 ± 14.8 years, suggesting a predominance among young to middle-aged adults, which may reflect higher transfusion exposure in reproductive or chronic disease populations. Among these patients, 49 (76.38%) were women and 16 (24.62%) were men, as shown in Table 1.

**Table 1:** Characteristics of alloantibody positive patients (n=65) Rh, Rhesus

Patient characteristic		Number (%)
Gender	Male	16 (24.62)
	Female	49 (76.38)
Age groups (in years)	1-15	5 (7.69)
	16-30	16 (24.61)
	31-45	21 (32.30)
	56-60	21 (32.30)
	61-75	2 (3.07)
	>75	0
ABO blood group	O	27 (41.54)
	A1	21 (32.31)
	A2	0
	B	11 (16.92)
	A1B	6 (9.23)
	A2B	0
Rh blood group	Rh D positive	52 (80)
	Rh D negative	13 (20)

Alloantibodies were most commonly detected in patients with blood group O (27/65, 41.54%), followed by A1 (21/65, 32.31%). Most alloantibodies were identified in patients aged 16 to 60 years (58/65, 89.23%).

As presented in Table 2, a single alloantibody was identified in 49 of 74 patients (66.21%), while seven of 74 (9.45%) had multiple alloantibodies. Cold autoantibodies were detected in nine of 74 patients (12.16%). The most frequently detected single alloantibody was anti-E (18/74, 24.32%), followed by anti-D (11/74, 14.86%).

**Table 2:** Distribution of RBC-directed unexpected antibody among the 74 patients Di, Diego; RBC, red blood cell

Patients with unexpected antibody (Anti-)			Number (%)
Single alloantibody	Rhesus system	D	11 (14.86)
		c	3 (4.05)
		E	18 (24.32)
		e	1 (1.35)
	Lewis system	Le^a^	2 (2.70)
		Le^b^	3 (4.05)
	Kidd system	Jk^a^	8 (10.81)
		Jk^b^	1 (1.35)
	Duffy system	Fy^b^	1 (1.35)
	MNS system	S	1 (1.35)
Multiple alloantibodies	D+C		2 (2.70)
	c+Di^a^		1 (1.35)
	E+Fy^b^		1 (1.35)
	E+Jk^a^		1 (1.35)
	Jk^a^+Le^b^		1 (1.35)
	Jk^b^+S		1 (1.35)
	Total (multiple)		7 (9.45)
Autoantibodies (cold)			9 (12.16)
Not determined			7 (9.45)
Total			74 (100)

**Figure 1:**
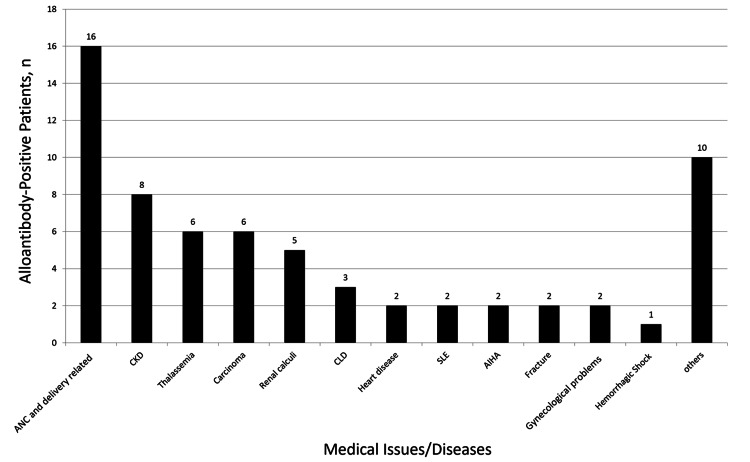
Distribution of alloantibody positive patients according to their medical information ANC, antenatal case; CKD, chronic kidney disease; CLD, chronic liver disease; SLE, systemic lupus erythematosus; AIHA, autoimmune hemolytic anemia

Among the 65 patients with alloantibodies, the highest number of cases were observed in antenatal patients (16/65, 24.61%), followed by individuals with chronic kidney disease (8/65, 12.30%). Alloantibodies were also detected in patients with thalassemia and various malignancies (6/65, 9.23% each), as illustrated in Figure 1.

All chronic kidney disease (CKD), thalassemia, and chronic liver disease (CLD) patients with alloantibodies had a past transfusion history. The youngest patient with alloantibodies, diagnosed with thalassemia, was four years old.

Among 49 alloantibody-positive female patients, nine (9/49, 18.36%) and 31 (31/49, 63%) had past pregnancy and RBC transfusion, respectively. Further, one female patient had both a past pregnancy and RBC transfusion history. Again, eight (8/49, 16.32%) female alloantibody-positive patients had no past pregnancy and RBC transfusion. Thirteen out of 16 (13/16, 81.25%) male alloantibody-positive patients had a past RBC transfusion history.

Table 3 shows the association between gender and alloantibody formation. Of the total study population, 4,358 (50.34%) were female individuals and 4,299 (49.65%) were male individuals. The odds ratio was 3.04 based on the chi-square test, indicating that female participants were three times more likely to develop alloantibodies than male participants.

**Table 3:** Association between frequency of alloantibody formation and gender (n=65) OR, odds ratio; CI, confidence interval

Gender	Presence of alloantibody		Prevalence of alloantibody (%)	Chi-square, χ^2^	P-value	OR	95% CI
	Yes	No					
Female	49	4309	1.12	16.43	<0.001	3.04	1.75-5.50
Male	16	4283	0.37				
Overall	65	8657	0.75				

Table 4 presents the relationship between prior PRBC transfusions and alloantibody formation. The chi-square analysis yielded an odds ratio of 2.95, suggesting that patients with a history of RBC transfusion were nearly three times more likely to develop alloantibodies than those without prior transfusions.

**Table 4:** Association between frequency of alloantibody formation and previous RBC transfusion (n=65) RBC, red blood cell; OR, odds ratio; CI, confidence interval

Previous RBC transfusion	Presence of alloantibody		Prevalence of alloantibody (%)	Chi-square, χ^2^	P-value	OR	95% CI
	Yes	No					
Yes	34	2327	1.44	20.69	<0.001	2.95	1.80-4.84
No	31	4283	0.49				

Table 5 shows the relationship between alloantibody formation, gender, and prior transfusion history. The highest prevalence (1.92%) of alloantibodies was found among women with a history of RBC transfusion, while the lowest prevalence (0.09%) occurred in men with no prior transfusion history.

**Table 5:** Relationship between alloantibody formation with gender and previous RBC transfusion (n=65) RBC, red blood cell

Gender	Previous RBC transfusion	Total patients, n	Presence of alloantibody, n	Alloantibody (%)
Female	Yes	1091	21	1.92
	No	3267	28	0.85
Male	Yes	1270	13	1.02
	No	3029	3	0.09

## Discussion

The overall prevalence of RBC alloantibodies in this study was 0.75%. The alloimmunization rate was higher in female participants (1.12%) than in male participants (0.37%). These findings are consistent with a study by Zaman et al., which reported an overall alloimmunization rate of 1.4%, with a higher prevalence among female patients (2.1%) than male patients (0.9%) [4]. In contrast, a study by Nathani et al. reported a lower prevalence of 0.28% [11]. Across various studies, reported alloimmunization rates range from 0.13% to 1.71% [1,4-9,11-14]. In our study, 49 of the 65 patients with alloantibodies (75.38%) were women. The higher rate in women may be attributed to antigenic exposure during pregnancy, a finding supported by previous studies [4,7,8,11].

We also observed a higher alloimmunization rate among patients with a history of RBC transfusion (1.44%) than those without prior transfusion (0.49%). This finding aligns with reports from other studies [7,14]. Our data demonstrate a significant association between prior transfusion and alloantibody development. Therefore, prophylactic antigen-matched blood transfusions should be considered for transfusion-dependent patients or those likely to receive multiple transfusions, such as individuals with thalassemia, sickle cell disease, cancer, or liver disease. These patients are at increased risk for alloimmunization, especially in regions where donor-recipient antigen mismatch is common [5].

Our study’s most frequently detected alloantibodies belonged to the Rhesus blood group system (33/65, 50.76%). Anti-E was the most frequently identified alloantibody (18/65, 24.32%), consistent with trends reported in other Indian and international cohorts. Anti-E is generally associated with mild, moderate, or rarely severe HDFN [15]. In contrast, a study by Nathani et al. reported anti-D as the most common antibody (19.17%) [11]. Rh (D) antigen is a potent immunogen, second only to ABO antigens, and is known to cause severe HDFN [15-17]. In our cohort, anti-D was present in 11 of 65 cases (14.86%). The 10.81% incidence of anti-Jka exceeds rates reported in other regions, possibly reflecting ethnic differences in Kidd antigen expression or transfusion practices in northeast India [5]. Kidd antibodies, particularly anti-Jka, can cause severe delayed hemolytic transfusion reactions [18].

Seven of 74 patients (9.45%) had multiple alloantibodies, complicating transfusion compatibility and increasing the risk for delayed hemolytic reactions. This may reflect the limitations of imported red cell panels, which may lack antigens present in the local population [14]. Most transfusion centers in India use antibody screening and identification panels manufactured in Western countries [11]. Salamat et al. emphasized the importance of using red cell panels derived from local populations to improve detection accuracy for indigenous antibodies [19].

Alloantibody development in patients with thalassemia, CKD, or CLD underscores the immunogenic potential of repeated transfusions, warranting extended phenotype matching in such populations. The presence of alloantibodies made it more difficult to find compatible blood units for these transfusion-dependent patients. Once alloantibodies are identified, both the treating physician and the patient should be informed. Patient records should be updated systematically to facilitate future transfusion management.

Our study’s most common clinical setting for alloantibody detection was among antenatal cases (16/65, 24.61%). This is likely due to maternal exposure to fetal antigens during pregnancy [20,21]. Antibody screening during antenatal visits is therefore crucial for the safety of both mother and fetus. Early detection facilitates timely intervention, ensures the availability of compatible blood units, and aids in diagnosing and managing HDFN.

Limitation

This study was limited by single-center design and lack of locally derived antigen panels. Future multicenter studies using indigenous cell panels are essential for a more accurate estimation of alloimmunization prevalence in the region. Secondly, parity data for female patients could not be retrieved, particularly for those seen in non-obstetric or non-gynecological settings.

## Conclusions

RBC alloimmunization was more frequent among women and patients with a history of prior RBC transfusions. The most commonly identified alloantibodies belonged to the Rhesus and Kidd blood group systems, with anti-E, anti-D, and anti-Jk^a^ being the most prevalent. Alloantibodies were most often detected in antenatal patients, highlighting the role of pregnancy as a sensitizing event.

Every hospital should have a hospital transfusion protocol in order to avoid unnecessary blood transfusions, especially for patients at increased risk of alloimmunization. Our region-specific findings support the implementation of targeted antigen-matching policies and the development of indigenous screening panels to enhance transfusion safety and reduce alloimmunization risk.
